# Sensitivity analysis of annual energy production and associated economic losses due to external wake effects on offshore wind clusters: A case study of the Shinan offshore wind cluster in southwest sea of Korea

**DOI:** 10.1371/journal.pone.0341955

**Published:** 2026-09-30

**Authors:** Geonhwa Ryu, Dohee Lee, Jungho Kim, Suji Han, Jun Yong Lee, Young-Gon Kim, Chae-Joo Moon

**Affiliations:** 1 Renewables Performance Team (Wind&Site), OWC, Seoul, Republic of Korea; 2 Jeonnam Offshore Wind Power Co., Ltd., Mokpo, Republic of Korea; 3 Independent Researcher, Republic of Korea; 4 Green Energy Institute, Mokpo, Republic of Korea; 5 KPGLab Co., Ltd., Naju, Republic of Korea; 6 Department of Energy Engineering, Korea Institute of Energy Technology, Naju, Republic of Korea; Wuhan University of Technology, CHINA

## Abstract

This study investigates the quantitative effects of external wake interactions and inter-farm spacing within a large-scale offshore wind cluster, focusing on the Shinan Cluster in Korea. As the country moves forward with government-led site development under the forthcoming Offshore Wind Special Act, the spatial clustering of multiple wind farms raises concerns about potential energy yield reductions and associated revenue losses caused by external wake effects. Using energy yield assessment simulations, a hypothetical 390 MW offshore wind farm was modelled adjacent to the Shinan offshore wind cluster. A sensitivity analysis was conducted across 12 scenarios by varying cluster completion levels (30%, 60%, and 100%) and inter-farm spacing distances (0 km, 1 km, 3 km, and 5 km). The results indicate that increasing the spacing between farms from 0 km to 3 km and 5 km reduced external wake losses from 8.0% to 5.2% and 4.3%, respectively, compared to the no-spacing case. For project developers, this translates into reductions in annual electricity revenue losses of approximately KRW 15.8 billion (3 km) and KRW 13.0 billion (5 km), based on a 2.5 REC scenario. These findings provide empirical evidence supporting the establishment of minimum inter-farm spacing guidelines to mitigate revenue loss and potential stakeholder conflicts within designated offshore wind cluster zones. The study offers actionable insights for policymakers and developers to promote sustainable offshore wind cluster design under Korea’s evolving regulatory framework.

## Introduction

As global efforts to combat climate change and strengthen energy security accelerate, offshore wind power has emerged as one of the most promising renewable energy sources [1,2]. Europe, led by Denmark, Germany, and the United Kingdom, has already accumulated extensive experience in operating offshore wind farms with capacities of several tens of gigawatts. More recently, large-scale offshore wind clusters have been actively developed and financed across East Asia, underpinned by ambitious national siting and development plans in countries such as China [3–5]. In Korea, the government has set forth the goal of achieving 18.3 GW of onshore and offshore wind capacity by 2030 under the 11th Basic Plan for Long-Term Electricity Supply and Demand. The national energy transition strategy increasingly prioritizes offshore wind deployment, with large-scale cluster developments planned and promoted in regions such as Shinan, Yeonggwang, and Gunsan in the southwestern seas [6].

Spatial development models for offshore wind farms in Korea can be classified into three categories: (1) the individually-developed sites led by private sector initiatives, (2) the cluster-type zones led by local governments that identify and plan sites exceeding 40 MW, and (3) the government-designated planned sites established under the legal framework of the forthcoming Offshore Wind Special Act [7,8]. The planned-site model represents a top-down national approach where optimal sites, permitting processes, and spatial coordination are managed by the central government. In contrast, the cluster-type zones function as a bottom-up model, initiated by local governments emphasizing community acceptance and regional participation. Designation as a cluster-type zone provides several policy advantages. It grants eligibility to request joint grid connection facilities from the Korea Electric Power Corporation (KEPCO), offers partial permitting support from central authorities, and may include financial incentives of up to 0.1 Renewable Energy Certificate (REC) credits per MWh for power generated within the zone. The resulting benefits can be redistributed to local governments and communities. These incentives enhance regional co-benefits while improving efficiency in grid connection, permitting, and environmental management. Since 2020, the Ministry of Trade, Industry, and Energy (MOTIE) and the Korea Energy Agency have implemented the “Public-Led Offshore Wind Development Support Program,” assisting local governments such as Gunsan, Boryeong, Taean, Shinan, Yeosu, Incheon, Pohang, and Goheung in developing large-scale offshore wind projects [8,9]. Consequently, 1.4 GW in Jeonbuk and 3.2 GW in Shinan were officially designated as offshore wind cluster zones by MOTIE in 2025. Other local governments, including Incheon and Boryeong, are also pursuing cluster designation in coordination with public–private working groups ahead of the Offshore Wind Special Act’s enforcement.

Despite these developments, current domestic regulations lack explicit technical criteria for determining the optimal spacing between individual offshore wind farms and do not provide guidelines for mitigating external wake losses which is a critical factor that influences both energy yield and developer profitability. While Korea’s policy framework emphasizes quantitative expansion through large-scale cluster or planned-site development [10,11], discussions concerning qualitative issues such as inter-farm energy losses, financial and economic impacts of external wakes, and conflict management among developers remain limited. There is also growing recognition of the need to define appropriate inter-farm spacing standards and spatial allocation criteria as government-led development expands. Experience from mature offshore wind markets highlights the importance of addressing these challenges. In Europe, for instance, external wake interactions between large-scale clusters and neighbouring wind farms have led to capacity factor reductions, revenue losses, and even legal disputes among project stakeholders. Cases such as the Hornsea development in the United Kingdom have brought to light compensation claims, spacing policy debates, and liability issues linked to wake-induced losses [8]. Furthermore, the legal dispute between Iberdrola and RWE concerning the East Anglia 2 and Five Estuaries projects, as well as the request to halt the Awel y Môr project due to insufficient pre‑assessment and mitigation of external wake losses, are also closely related to these “wind theft” issues [8].

Existing studies have demonstrated that neighbouring‑farm wakes can persist over tens of kilometres offshore, reduce capacity factors of downwind projects, and in some cases give rise to disputes over liability and compensation. However, most of this work focuses on the North Sea and other European waters, and very few studies quantify how such cluster‑scale wakes translate into long‑term AEP and financial metrics under the specific regulatory and market conditions of emerging offshore wind markets such as Korea.

To address this research gap, this study introduces a hypothetical 390 MW offshore wind farm within the Shinan Offshore Wind Cluster to quantitatively evaluate the effects of external wakes and inter-farm spacing. A total of 12 sensitivity scenarios were analysed, combining variations in cluster operation ratios (30%, 60%, and 100%) and inter-farm spacing distances (0 km, 1 km, 3 km, and 5 km). The analysis quantifies both the energy yield reduction caused by external wakes and the corresponding economic loss, taking into account Korea’s revenue structure under the System Marginal Price (SMP) and REC mechanisms.

Given the ongoing legal and institutional evolution of offshore wind policy, these results provide practical reference data for governments, regulators, and developers seeking to establish spatial design criteria for planned offshore wind zones. By objectively quantifying energy losses and economic impacts, the study underscores the necessity of introducing minimum spacing and wake management guidelines within clustered offshore wind developments. Such insights can contribute to preventing policy disputes, promoting sustainable industry growth, and supporting evidence-based planning for future large-scale offshore wind expansion in Korea.

## Literature review: External wake effects and inter-farm spacing

### External wake effects

As offshore wind clusters and government-led planned sites continue to expand, the issue of external wake interactions among adjacent wind farms is becoming increasingly significant [12–14]. In general, a wake refers to a flow phenomenon characterized by velocity deficit and enhanced turbulence that forms downstream of a wind turbine as it extracts kinetic energy from the wind [15]. The wake released from upstream turbines can negatively influence the performance of downstream turbines, leading to reduced power generation efficiency [16].

This phenomenon occurs not only within a single wind farm (internal wakes) but also between neighbouring farms within cluster zones, where its impact is amplified as farm sizes increase and spatial layouts become denser. Wake effects typically manifest as diminished wind velocity, increased turbulence intensity, and subsequent energy losses, all of which ultimately reduce the annual energy production (AEP) and financial performance of downstream assets [17–20]. Therefore, quantifying external wake effects and identifying optimal inter-farm spacing are essential for developers, policymakers, and regulators during the design and planning stages of offshore wind clusters.

### Policy and spatial planning frameworks for inter-farm spacing in offshore wind clusters

Inter-farm spacing serves as a key spatial design parameter for large-scale offshore wind developments organized through clustered or government-planned siting schemes. Adequate spacing between neighbouring wind farms helps minimize generation losses due to external wakes, ensures long-term energy efficiency, and reduces the risk of conflicts among project developers. Inadequate spacing can lead to wake-induced efficiency decline, economic loss, and even legal disputes between adjacent projects [21–23].

To mitigate such risks, many countries have established policies or engineering-based guidelines governing inter-farm spacing. In the United Kingdom, for instance, the Round 4 Offshore Wind Leasing guidelines specify a minimum distance of 7.5 km between new projects and at least 5 km from existing adjacent projects [8]. These regulations aim to minimize wake-induced energy losses and ensure safe marine navigational corridors. “Taiwan’s Offshore Wind Power Development Rules” legally mandate a minimum spacing of 1.2 km between offshore wind farms.

In contrast, countries such as Germany, the Netherlands, and Denmark have not set fixed numerical criteria for inter-farm spacing. Instead, spacing and layouts are determined within the context of Marine Spatial Planning (MSP) processes, which consider navigational safety, environmental preservation, and other competing ocean uses in an integrated manner.

Currently, Korea lacks explicit legal criteria concerning the minimum spacing between offshore wind farms to mitigate external wake effects. The Offshore Traffic Safety Assessment Guidelines prescribe separation distances based primarily on navigational safety defined as six times the length of the largest passing vessel plus 500 m and 0.3 nautical miles [24]. These criteria prioritize maritime safety rather than energy production efficiency or wake loss mitigation.

Considering the nation’s transition toward government-led planned sites and large-scale cluster-type developments, there is an urgent need to establish a formal regulatory framework for inter-farm spacing. Incorporating international policy best practices and scientific research findings into Korean spatial planning policies would help prevent energy yield and revenue losses due to external wakes. Moreover, such institutionalization would promote sustainable industry growth and facilitate equitable coordination among offshore wind developers in the long term.

## Experimental setup and methodology

### Study area

According to data from the Electricity Regulatory Commission of the MOTIE as of September 2025, a total of 109 offshore wind projects in Korea have received energy business licenses (EBL), amounting to an aggregate authorized capacity of approximately 34.8 GW (excluding cancelled and relinquished permits) (Table 1). Over 60% of these projects are concentrated in Jeonnam Province, where 68 projects with a combined capacity of around 21.3 GW have been approved (Fig 1), establishing the region as the nation’s focal point for offshore wind development. Ulsan and Incheon are also advancing large-scale offshore wind clusters, with project pipelines totalling approximately 6.0 GW and 3.4 GW, respectively, indicating the nationwide proliferation of offshore wind cluster development.

**Table 1:** Status of offshore wind projects which has secured offshore wind energy business licenses by region in South Korea as of September 2025.

Region	Number of Projects	Total Planned Capacity [MW]	Regional Proportion [%]
Gyeonggi	1	200	0.6
Gyeongnam	4	926	2.7
Gyeongbuk	1	96	0.3
Busan	2	136	0.4
Ulsan (2nd)	13	5,961	17.1
Incheon (3rd)	8	3,415	9.8
Jeonnam (1st)	68	21,339	61.3
Jeonbuk	3	529	1.5
Jeju	4	307	0.9
Chungnam	5	1,929	5.5
Total	109	34,838	100.0

**Fig 1:**
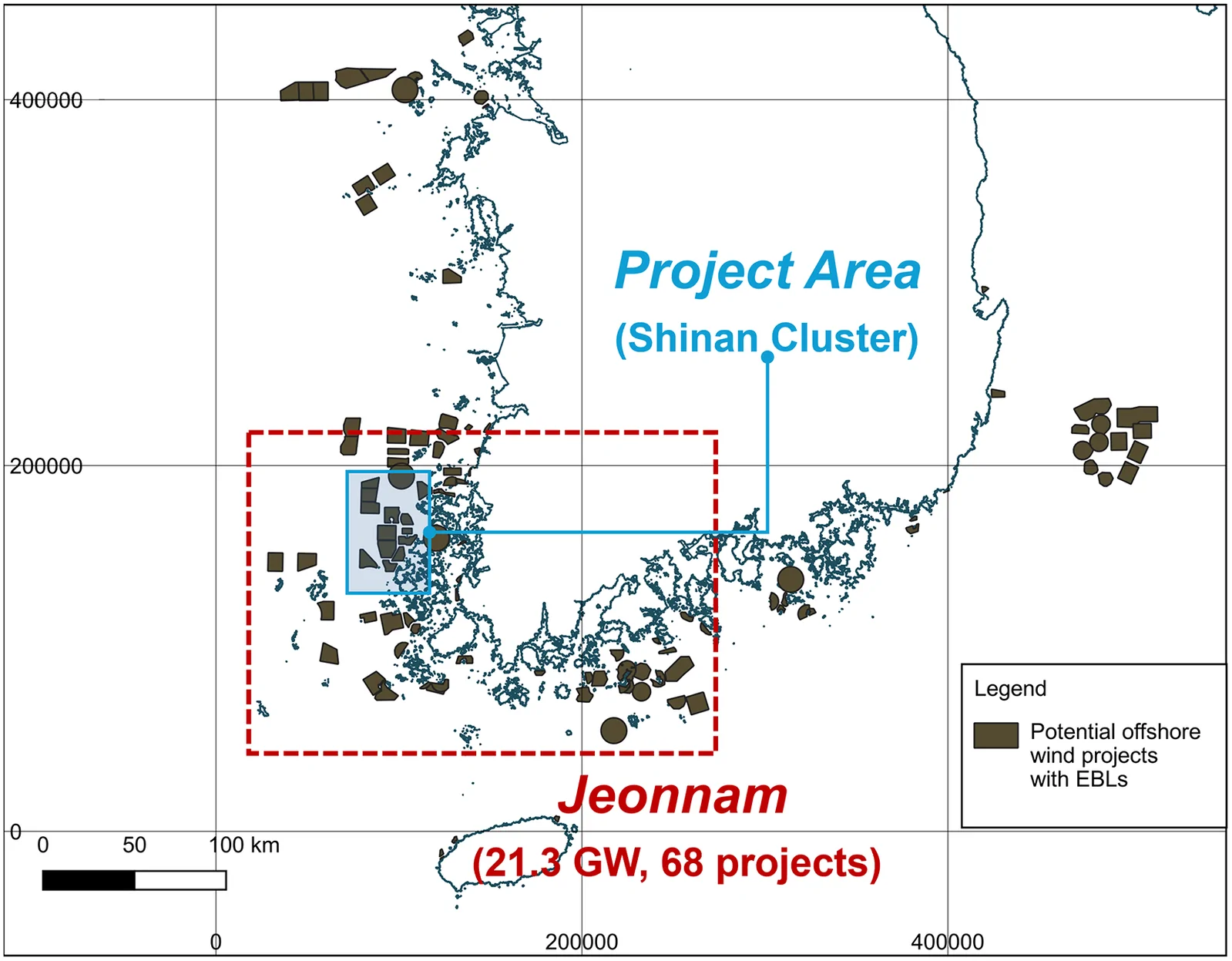
The spatial distribution of offshore wind projects in South Korea which have secured energy business licenses, visualized using QGIS. The blue square represents the Shinan offshore wind farm cluster. Coordinate system: EPSG 5181, WGS 84 / Pseudo-Mercator.

Of particular note, Shinan County in Jeonnam was officially designated as a renewable energy cluster zone in April 2025 by MOTIE, encompassing ten cluster-type projects with a total capacity of 3,166 MW. Accordingly, this study selects the Shinan offshore wind cluster zone as a representative case to quantitatively analyse energy yield losses resulting from external wake interactions under large-scale clustered deployment scenarios.

### Simulation of external wake effects on cluster zones

This study quantitatively evaluates energy yield losses caused by external wake effects within selected cluster-type offshore wind zones in Shinan County, Jeonnam Province. To achieve this, a hypothetical offshore wind project area was established adjacent to the Shinan cluster zone, and wind turbines were spatially arranged within both the cluster and the hypothetical project site. The turbine layout was based on the open-source 15 MW reference wind turbine model developed by the IEA [25–27], which was applied to both the cluster-type zones and the hypothetical project to closely replicate real-world development scenarios.

The installed capacity and number of turbines for each zone were determined according to local characteristics, using an average energy density criterion of approximately 5.0 MW/km² for the cluster-type zones. The hypothetical project was designed using a higher density criterion of roughly 9.0 MW/km², resulting in a total capacity of 390 MW to emphasize wake effects. Considering the prevailing wind direction in the southwest sea (northwest–north-northwest), the hypothetical project wind farm was positioned as far downstream as possible relative to the cluster, to conservatively assess maximum wake impacts. The design capacity for each of the ten cluster zones (Zones 1–10) was calculated using the energy density criterion (see Fig 2, Table 2).

**Table 2:** Basic assumptions and wind farm layout design for each subarea (No. 1–10) and a hypothetical project site of the Shinan offshore wind farm cluster. The long-term wind speed at hub height (150 m) is extracted from the Vortex mesoscale modelled wind resource map.

Site No.	Site area [km2]	Wind farm capacity [MW]	No. of WTGs	Wind farm density [MW/km2]	Long-term average wind speed at hub height [m/s]	Rotor diameter & Hub height [m]	WTG spacing	
							Prevailing [RDs]	Cross wind [RDs]
1	67.9	345	23	5.1	7.51	240 / 150	10.0	5.5
2	69.4	360	24	5.2	7.53	240 / 150	9.0	5.5
3	66.0	345	23	5.2	7.55	240 / 150	9.5	5.5
4	65.4	345	23	5.3	7.58	240 / 150	9.5	5.5
5	67.9	345	23	5.1	7.58	240 / 150	9.5	5.5
6	42.1	225	15	5.3	7.61	240 / 150	9.5	5.0
7	76.0	390	26	5.1	7.19	240 / 150	9.0	5.5
8	33.8	165	11	4.9	7.41	240 / 150	10.0	6.0
9	67.3	315	21	5.3	7.49	240 / 150	9.5	5.5
10	65.0	345	23	5.1	7.48	240 / 150	9.5	5.0
Project Site	43.1	390	26	9.1	7.33	240 / 150	7.0	5.0

**Fig 2:**
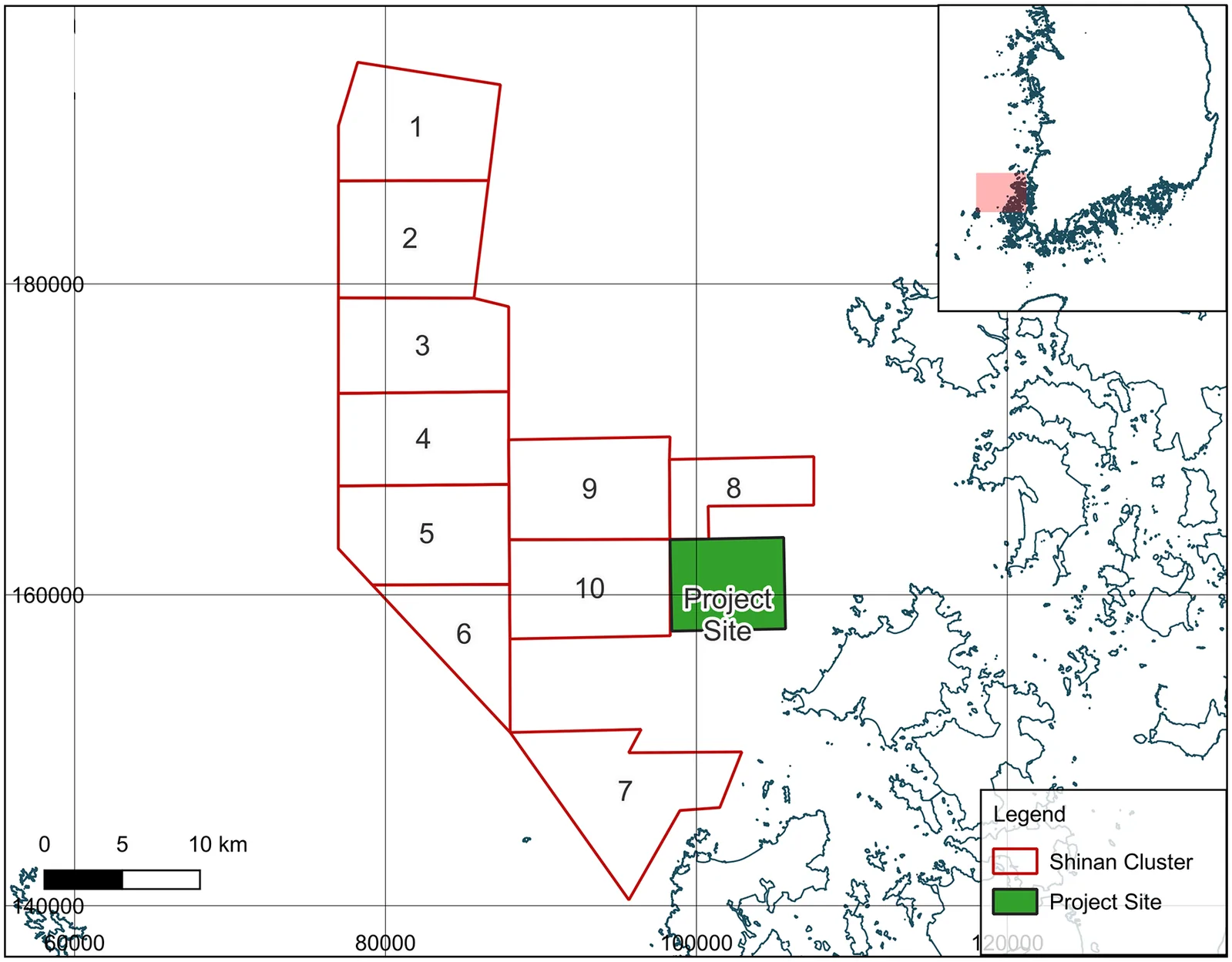
Assumed arbitrarily located offshore wind project site (green shaded area) and the Shinan offshore wind cluster (red boundary zones of No. 1–10) for external wake simulation in this study. Coordinate system: EPSG 5181, WGS 84 / Pseudo-Mercator.

The overall analysis procedure is as follows (Fig 3):

**Fig 3:**
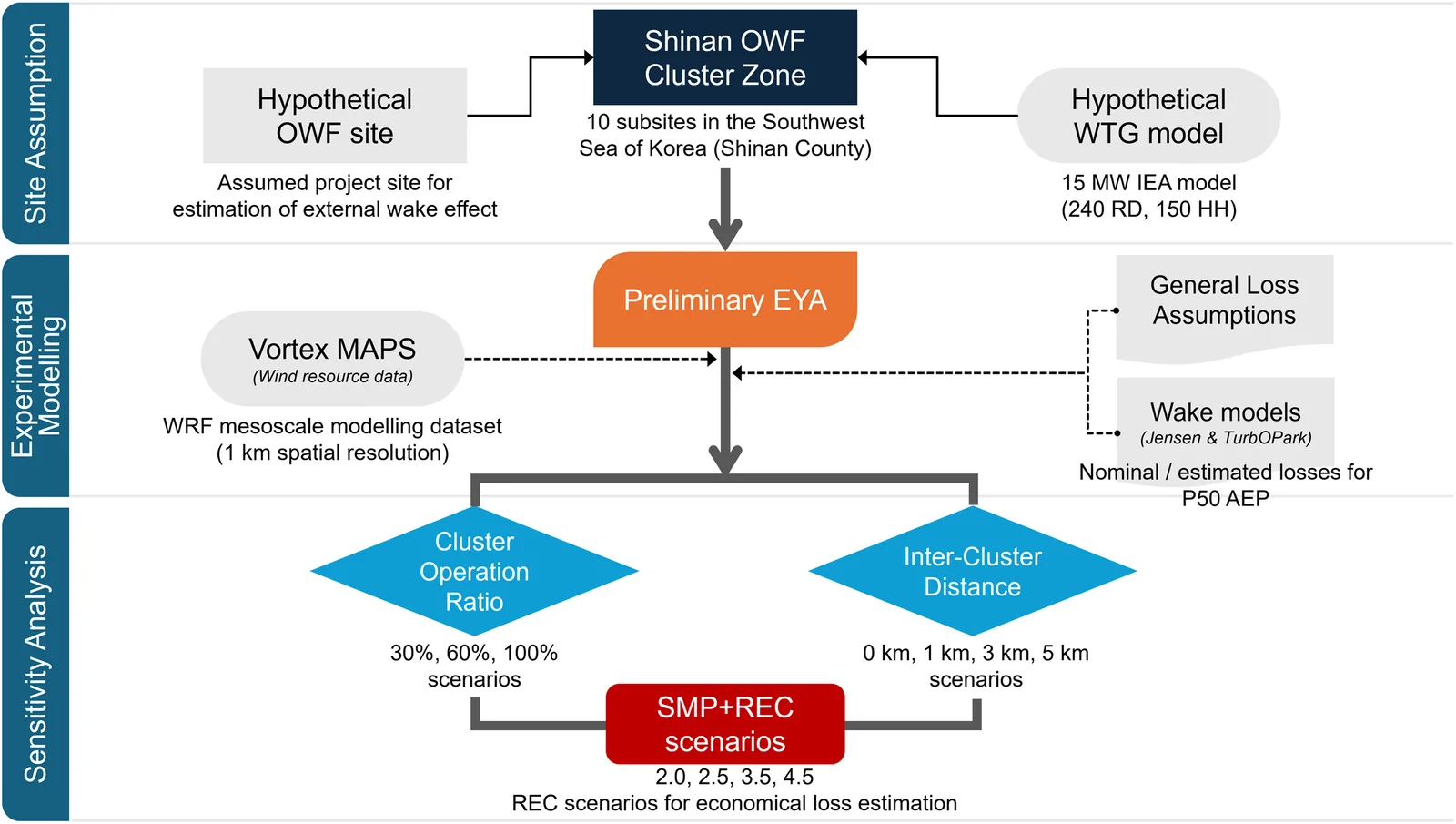
Analytical procedure for sensitivity analysis of annual economic loss due to external wakes in the Shinan offshore wind cluster zone, Southwest Korea.

- A hypothetical offshore wind farm was placed within the Shinan cluster zone in Jeonnam.
- Both the cluster-type zones and the hypothetical farm were designed using the 15 MW reference wind turbine model.
- Three scenarios were constructed based on the completion and operational ratio of each cluster project area (30%, 60%, 100%).
- Four scenarios were established by varying the inter-farm spacing between project zones (no spacing, 1 km, 3 km, 5 km).
- Combining cluster operation ratios and inter-farm spacing, a total of 12 cases were used to perform sensitivity analyses of wake-induced energy yield losses.
- For each case, economic loss sensitivity analyses were also performed at various REC multipliers (2.0, 2.5, 3.5, 4.5 REC) based on the latest SMP + REC market conditions.

As a result, AEP losses and associated economic losses were estimated for each scenario. This allowed for a quantitative assessment of the business impacts of external wake effects and the formulation of relevant policy implications for offshore wind cluster development.

### Wind resource input data

Vortex MAPS mesoscale modelling data was utilized as the primary wind resource input. Vortex MAPS, based on the Weather Research and Forecasting (WRF) [28] numerical weather model, is specifically designed for regional wind resource assessment and leverages reanalysis datasets such as ERA5 to generate long-term averages for wind speed, turbulence intensity, and vertical/horizontal profiles over periods exceeding 20 years with a horizontal resolution of 1 km [29]. For this analysis, Vortex MAPS provided long-term wind resource characteristics including mean wind speed, direction, temperature, and turbulence intensity for each candidate site. Most notably, Vortex MAPS serves as an alternative data source reflecting long-term climate characteristics, making it suitable for spatial distribution and vertical profile analysis during the early design stages of large offshore wind cluster developments.

## External wake sensitivity analysis

### Cluster operation ratios

Considering that individual sites within cluster zone 1–10 may not be completed and operated simultaneously, and recognizing that not all sites may progress as planned, the external wake loss sensitivity scenarios were established based on the operational ratio of the 10 cluster sites as follows:

- Scenario Base: No external wake losses present (only the hypothetical wind farm is operational)
- Scenario 1: 30% of cluster projects (3 sites, selected randomly) are completed
- Scenario 2: 60% of cluster projects (6 sites, selected randomly) are completed
- Scenario 3: 100% of cluster projects (all 10 sites) are completed

### Adjustment of inter-cluster distances

Sensitivity scenarios for adjusting distances between the hypothetical project area and each cluster site (1–10) were devised. Minimum inter-cluster distance guidelines such as Taiwan’s 1.2 km and the UK’s 5.0–7.5 km were referenced, and four representative distances (0 km, 1 km, 3 km, and 5 km) were adopted for the analysis:

- Scenario 1: No inter-cluster distance is applied (clusters are contiguous)
- Scenario 2: All cluster distances set to 1 km
- Scenario 3: All cluster distances set to 3 km
- Scenario 4: All cluster distances set to 5 km

### Final sensitivity analysis scenarios

Table 3 summarizes the final sensitivity analysis scenarios. The “Base case” assumes that only the hypothetical project is sited, and no external wake-induced energy losses occur. Scenarios range from Case 1 (no spacing inter-cluster with 30% operation) to Case 12 (5 km inter-cluster distance with 100% cluster operation), evaluating annual energy yield across 12 scenarios. Quantitative estimation of energy and economic losses for Cases 1–12 is provided relative to the Base case, which considers losses from internal wake effects only. The results of wind farm and turbine layouts for each scenario are shown in Fig 4.

**Table 3:** Sensitivity analysis scenarios based on cluster operation ratios and inter-cluster distance (wind farm spacing). The base case represents only internal wake loss without considering any neighboring offshore wind farms.

Scenario		Cluster Operation Ratios			
		0%	30%	60%	100%
Inter-Cluster distance	No buffer	Base case	Case 1	Case 2	Case 3
	1 km		Case 4	Case 5	Case 6
	3 km		Case 7	Case 8	Case 9
	5 km		Case 10	Case 11	Case 12

**Fig 4:**
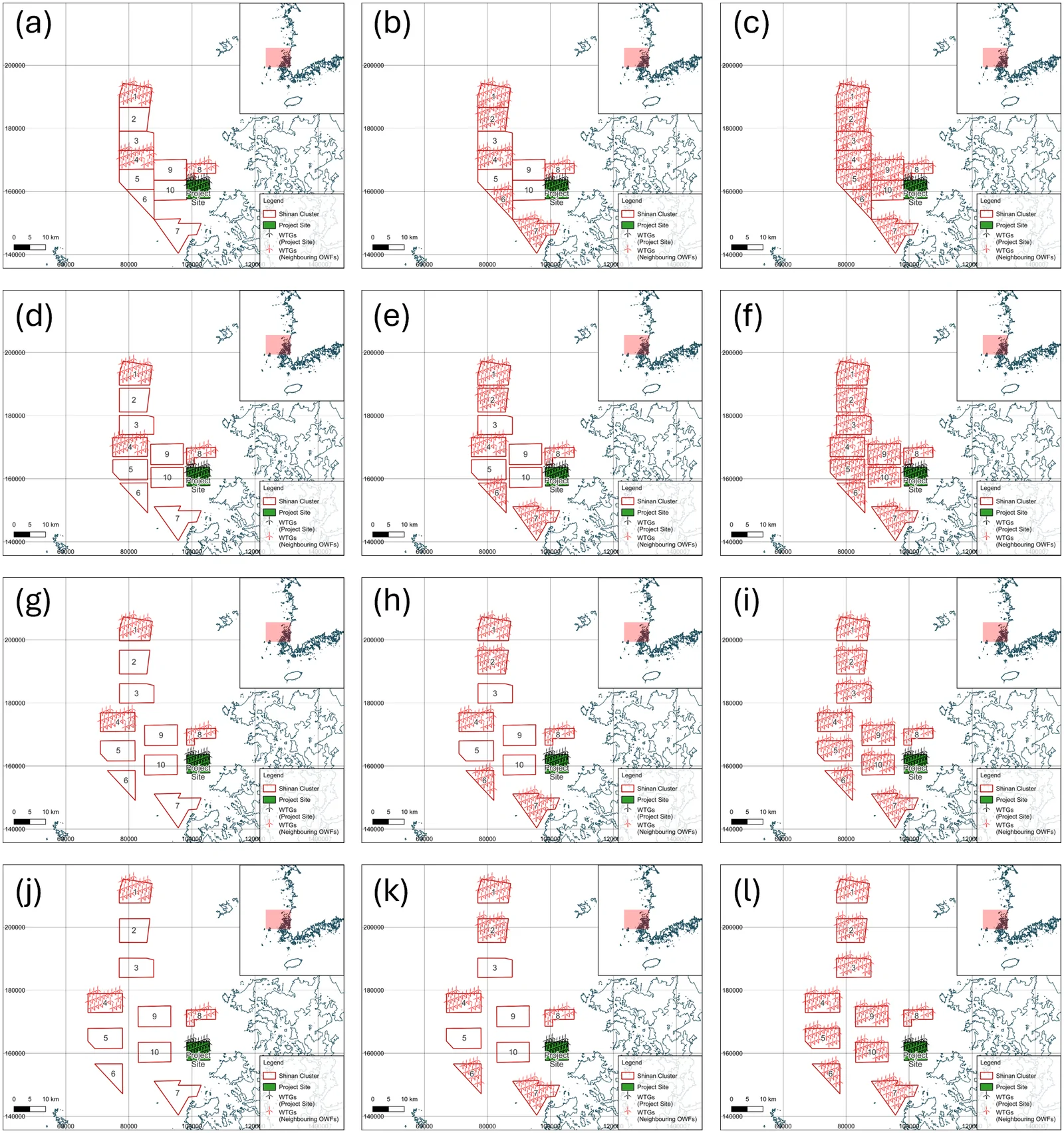
Configuration of external wake scenarios considering wind farm cluster operation ratios and inter-cluster distance (wind farm spacing): (a) Case 1, (b) Case 2, (c) Case 3, (d) Case 4, (e) Case 5, (f) Case 6, (g) Case 7, (h) Case 8, (i) Case 9, (j) Case 10, (k) Case 11 and (l) Case 12. Coordinate system: EPSG 5181, WGS 84 / Pseudo-Mercator.

## Wake models

### Jensen wake model

Commonly known as the Park model, the Jensen wake model is an analytical framework that linearly simplifies and predicts far-wake wind speed deficits occurring downstream of wind turbines (see Equation 1) [30,31]. The model is based on the assumption that the low-speed, high-turbulence zone formed behind a turbine due to energy extraction diffuses linearly with distance, resulting in gradual wind speed recovery.


$$\begin{array}{c} U(x)=\;U_{\text{0}}\left(\text{1}-\frac{\text{1}-\sqrt{\text{1}-C_{t}}}{(\text{1}+\text{2}kx/D{)}^{\text{2}}}\right)\;[m/s] \end{array}$$ (1)

In this context, U(x)  represents the relative wind speed at a downstream distance x from the turbine, while $U_{\text{0}}$ denotes the free-stream velocity upstream of the turbine. $C_{t}$ refers to the thrust coefficient, and k is the wake decay constant (WDC). For offshore conditions, a WDC value of 0.04–0.06 is typically applied; this study adopts a representative value of 0.04. D denotes the rotor diameter of the turbine.

For each wind speed–direction bin ($U_{m},\;\theta_{n}$), the single-wake velocity deficit from an upstream turbine j at the location of turbine i is first expressed as


$$\begin{array}{c} \delta_{j\to i}=\;\frac{U_{\text{0}}-U_{j\to i}}{U_{\text{0}}} \end{array}$$ (2)

where $U_{\text{0}}$ is the free‑stream wind speed at hub height and $U_{j\to i}$ is the local wind speed at turbine i when only the wake from turbine j is considered.

The combined velocity deficit at turbine i due to multiple upstream turbines is then obtained using the root‑sum‑square superposition method as below:


$$\begin{array}{c} \delta_{i}=\;\sqrt{\sum_{j\in u(i)}\delta_{j\to i}^{\text{2}}} \end{array}$$ (3)

where u(i) denotes the set of all turbines located upstream of turbine i for the considered wind direction.

The effective hub‑height wind speed at turbine i is thus given by:


$$\begin{array}{c} U_{i}=\;U_{\text{0}}(\text{1}-\delta_{i}) \end{array}$$ (4)

The Jensen model is widely used in commercial software as a default wake model, valued for its computational simplicity and flexible parameterization, making it applicable across both offshore and onshore contexts. However, its limitations include inadequate representation of turbulence intensity changes, inter-turbine interactions in large arrays, nonlinear wind speed distribution, and atmospheric effects. Consequently, it is known to have accuracy limitations and a tendency to underestimate wake losses in practical wind farm applications.

### TurbOPark wake model

Unlike the Jensen model, which assumes linear wake expansion, the TurbOPark (Turbulence Optimised Park) model accounts for wake expansion deceleration as turbulence generated within the wake dissipates with increasing distance [32–35]. TurbOPark complements the physical interpretation of inter-turbine interactions by more accurately reflecting nonlinear effects such as wind speed deficits, turbulence intensity, and thermal impacts when multiple wakes overlap. This approach enables the prediction of induced losses not only for single turbine wakes but also for complex aggregated scenarios within large wind farms [36–39].

In this study, the TurbOPark model as implemented in the WindPRO PARK module is used to represent far‑wake development and inter‑farm interactions. The model retains the top‑hat assumption of the Jensen/Park approach but links the wake expansion rate to the local turbulence intensity, allowing the wake to recover faster in highly turbulent conditions while remaining persistent in low‑turbulence offshore environments.


$$\begin{array}{c} R(x)=\;\frac{D}{\text{2}}+k_{TP}x \end{array}$$ (5)

$$\begin{array}{c} {\Delta U}_{j\to i}=\;U_{\text{0}}\left[\text{1}-\sqrt{\text{1}-\frac{C_{T}}{\text{8}{\left(\frac{R(x)}{D/\text{2}}\right)}^{\text{2}}}}\right] \end{array}$$ (6)

The single‑wake deficits obtained from Equation (6) are mapped to the turbine locations and combined using the root‑sum‑square superposition described in Equation (3), yielding the effective hub‑height wind speed at each turbine before converting to power and AEP.

The formulation of the TurbOPark model builds upon the Jensen framework while introducing probabilistic and weighting methods to quantify the cumulative wake effects from multiple turbines downstream [40]. This model is particularly suitable for simulating external wake effects in large clusters, although it may conservatively estimate the magnitude and persistence of offshore external wake influences, potentially resulting in conservative annual energy yield predictions.

### Selection of wake models for external wake assessment

For the analysis of external wake losses in offshore wind cluster developments, both the Jensen and TurbOPark wake models were selected, and an ensemble modelling approach was adopted by equally weighting the results from each model (weighting factor of 0.5). This methodology accounts for the inherent bias tendencies of each model: the Jensen model’s tendency to oversimplify wake expansion and thus underestimate wake losses, and TurbOPark’s propensity to overestimate losses by capturing nonlinear cumulative wake effects. To address these contrasting characteristics, model outputs were independently derived and then integrated with equal weights to arrive at the final loss estimate.

Meanwhile, the Eddy Viscosity (EV) wake model was excluded from consideration, as it requires detailed atmospheric turbulence and flow field data not available during the early-stage, pre-feasibility assessment targeted in this project. The EV model is subject to considerable uncertainty in the absence of ample site measurements and detailed flow parameters, and its computational complexity limits practicality for large offshore wind cluster early-phase reviews [40,41]. Consequently, it was excluded from the model shortlist. Table 4 presents internal wake loss simulation results for the 390 MW project area under the Base case scenario (internal wake effects only) using the selected models.

**Table 4:** The summary result of preliminary EYA simulation for internal wake losses including blockage effect. The adjusted final wake loss was estimated from Jensen and TurbOPark as an ensemble methodology considering same weighting value.

Scenario	Wake model	Internal wake losses [%]	Weighting value	Adjusted wake losses [%]
Base case (considering only internal wake)	Jensen (Park)	11.91	50%	12.43
	TurbOPark	12.96	50%	
	Eddy Viscosity (EV)	9.51	–	–

Due to its tendency to underestimate internal wake loss, the Eddy Viscosity model was also excluded from the ensemble methodology. The final internal wake loss estimate was derived by applying a weighting factor of 0.5 to each of the Jensen and TurbOPark model results. This ensemble approach was also consistently applied to simulations of external wake effects.

## Results and discussion

### Core assumptions for energy yield assessment

The P50 energy yield represents the 50th percentile of long-term average energy production, meaning there is a 50% probability that actual generation will meet or exceed the predicted value. The P50 figure is routinely used for project feasibility evaluations, profitability analyses for investors, and credit/bond assessments by financial institutions, often serving as the ‘Best Case’ scenario during feasibility studies [6]. More conservative risk evaluations such as P75 and P90 percentiles are also commonly calculated, but P50 remains the technical and economic decision-making baseline.


$$\begin{array}{c} {AEP}_{P\text{5}\text{0}}=\;{AEP}_{gross}\times (\text{1}-{Losses}_{Total}[\%]) \end{array}$$ (7)

P50 is determined by predicting the long-term energy yield of a wind project, incorporating a range of loss factors (Equation 7). These loss factors include wake losses (turbine interactions), electrical losses, mechanical or turbine availability losses, environmental losses, curtailment (grid restrictions), non-availability due to maintenance and inspections, and other exceptional losses [42,43]. Each loss factor is applied multiplicatively after the theoretical annual energy production (Gross AEP) is calculated, producing the ‘Net AEP’, the P50 yield expressed from a probabilistic perspective. In this study, loss rates assumed for all factors except wake losses are shown in Table 5.

**Table 5:** The breakdown of general loss assumptions excluding turbine interactions (wake losses).

Subcategories of secondary losses	Assumed value	
	Losses [%]	Efficiencies
Non-availability	5.4	0.946
Electrical efficiency	3.5	0.965
WTG performance	1.7	0.983
Environmental	0.6	0.994
Curtailment	0.0	1.000
Total	10.8	0.892


$$\begin{array}{c} {Losses}_{Total}=\text{1}-\left[\left(\text{1}-{Loss}_{A}\right)\times \left(\text{1}-{Loss}_{B}\right)\times \cdots \times \left(\text{1}-{Loss}_{N}\right)\times \right] \end{array}$$ (8)

Based on equation 8, the loss rate excluding wake losses is set at 10.8%, which is considered a suitable benchmark in the context of domestic environmental conditions [6]. The P90/P50 ratio serves as a supplementary indicator for rapid estimation of P90 yields when detailed uncertainty analysis is unavailable [44]. This ratio reflects the spread of project energy yield distribution; values close to 1 indicate low prediction uncertainty. Generally, offshore wind projects exhibit lower resource variability than onshore sites, allowing P90/P50 ratios of 0.87–0.93 to be considered commercially sound, indicating that loss and uncertainty are at an acceptable level for reliable investment and financial review.

Given the absence of dedicated wind resource measurements in this study, an independent uncertainty analysis could not be performed. Therefore, the P90/P50 ratio was presumed to be 0.9 for P90 yield estimation.

### Wind speed deficit

Figs 5 and 6 illustrate the spatial variability of wake-induced wind speed deficits within the Shinan offshore wind cluster under various inter-farm spacing scenarios, where 11 individual sites (10 in red delineated cluster areas plus 1 project site outlined in dashed black) are assumed fully outfitted and operational with turbines. The scenarios model wind farm spacing at 0 km (Case 3), 1 km (Case 6), 3 km (Case 9), and 5 km (Case 12) to investigate the influence of cluster layout density on wake interactions and resultant wind speeds at turbine hub height (150 m).

**Fig 5:**
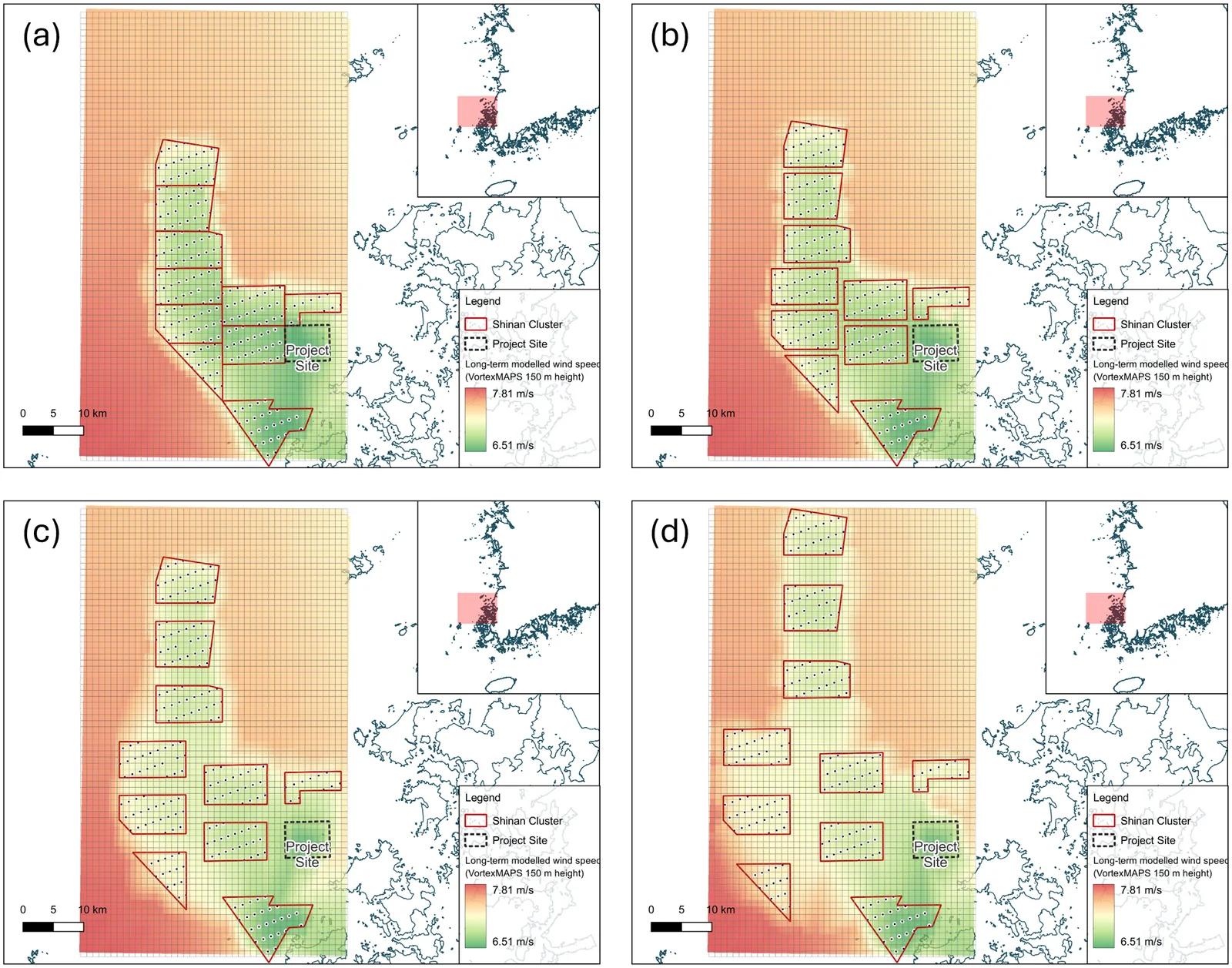
Quantitative distribution of hub-height (150 m) wind speed deficits induced by internal/external wakes in the Shinan cluster under different scenarios. All scenarios assume 100% operational ratio of the cluster, with inter-farm spacing varied across the following cases: (a) Case 3 − 0 km, (b) Case 6 − 1 km, (c) Case 9 − 3 km, and (d) Case 12 − 5 km. Coordinate system: EPSG 5181, WGS 84 / Pseudo-Mercator.

**Fig 6:**
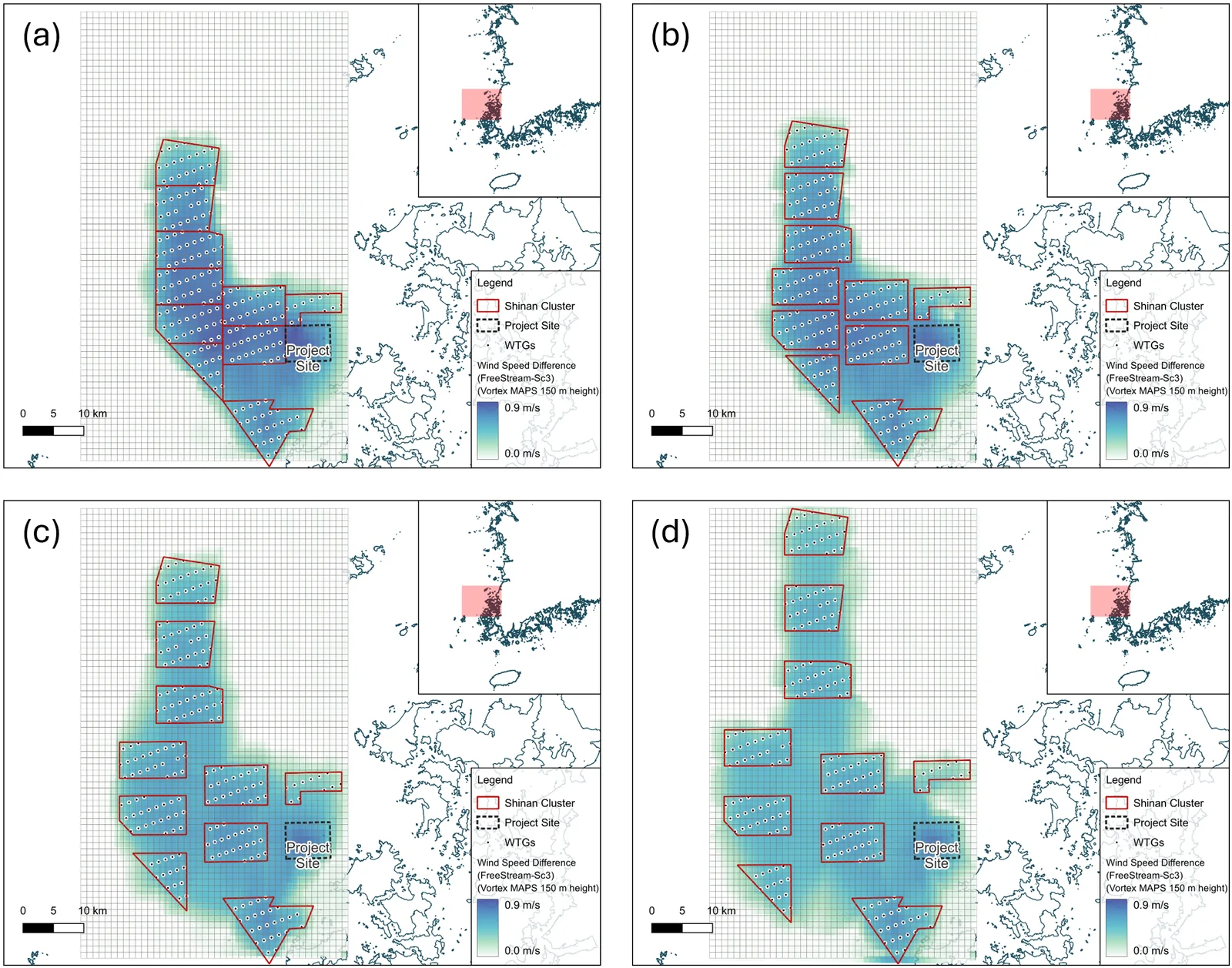
Quantitative distribution of wind speed difference at hub-height (150 m) between free wind stream and wake-effected wind speed induced by internal/external wakes in the Shinan cluster under different scenarios. All scenarios assume 100% operational ratio of the cluster, with inter-farm spacing varied across the following cases: (a) Case 3 − 0 km, (b) Case 6 − 1 km, (c) Case 9 − 3 km, and (d) Case 12 − 5 km. Coordinate system: EPSG 5181, WGS 84 / Pseudo-Mercator.

Fig 5 presents the Vortex MAPS mesoscale modelling wind resource dataset depicting the spatial distribution of wind speeds at hub height for each spacing scenario. The colour gradient from red to green indicates decaying wind speeds due to wake effects, with green corresponding to greater deficits. Notably, Case 3 (0 km inter-farm spacing) demonstrates extensive areas of pronounced wind speed reduction, particularly near turbine clusters, as evidenced by the deep green shading. This intensified velocity deficit arises from overlapping wakes caused by the close proximity of wind farms, producing compounded aerodynamic interference, increased turbulence intensity, and subsequent momentum depletion in the wake region.

Fig 6 visualizes the quantitative wind speed difference between free-stream undisturbed winds (turbine-free conditions) and wake-affected winds within the same cluster scenarios. The blue shading reflects the magnitude of wind speed deficit attributable to wake effects, with darker blue regions indicating a deficit of up to 0.9 m/s relative to free-stream conditions. As in Fig 5, Case 3 exhibits the most extensive and severe velocity deficits, illustrating substantial wake-induced wind speed reduction when turbine arrays are densely packed without spatial separation.

Increasing inter-farm spacing from 0 km to 5 km progressively mitigates these wake interactions, as the additional distance allows greater wake recovery and dispersion before encountering subsequent turbines. This spacing improvement is manifested by weakening green and blue regions in Figs 5 and 6 for Cases 6, 9, and 12, indicating higher hub-height wind speeds and smaller wake-induced deficits.

Fig 7 further illustrates the cross-sectional spatial variation of wind speed deficit across the Shinan cluster. It is evident that the magnitude of the difference between the free-stream wind speed and the waked wind speed progressively increases toward the central region of the cluster. This trend is attributable to the cumulative effect of overlapping wakes generated by upstream turbine arrays, which become more pronounced as the wind progresses into the denser core of the cluster. Furthermore, a clear distinction is observed among the different spacing scenarios: as the inter-farm spacing increases from Case 3 to Case 12, the severity of the wake-induced wind speed reduction is markedly alleviated. This finding demonstrates that greater spacing between wind farm sub-clusters effectively mitigates the adverse impact of wake losses on the available wind resource, thereby reducing the expected negative effect on annual energy production. These results underscore the importance of optimized spatial planning for large-scale offshore wind clusters, ensuring that wake interactions are minimized to maximize power yield and operational efficiency.

**Fig 7:**
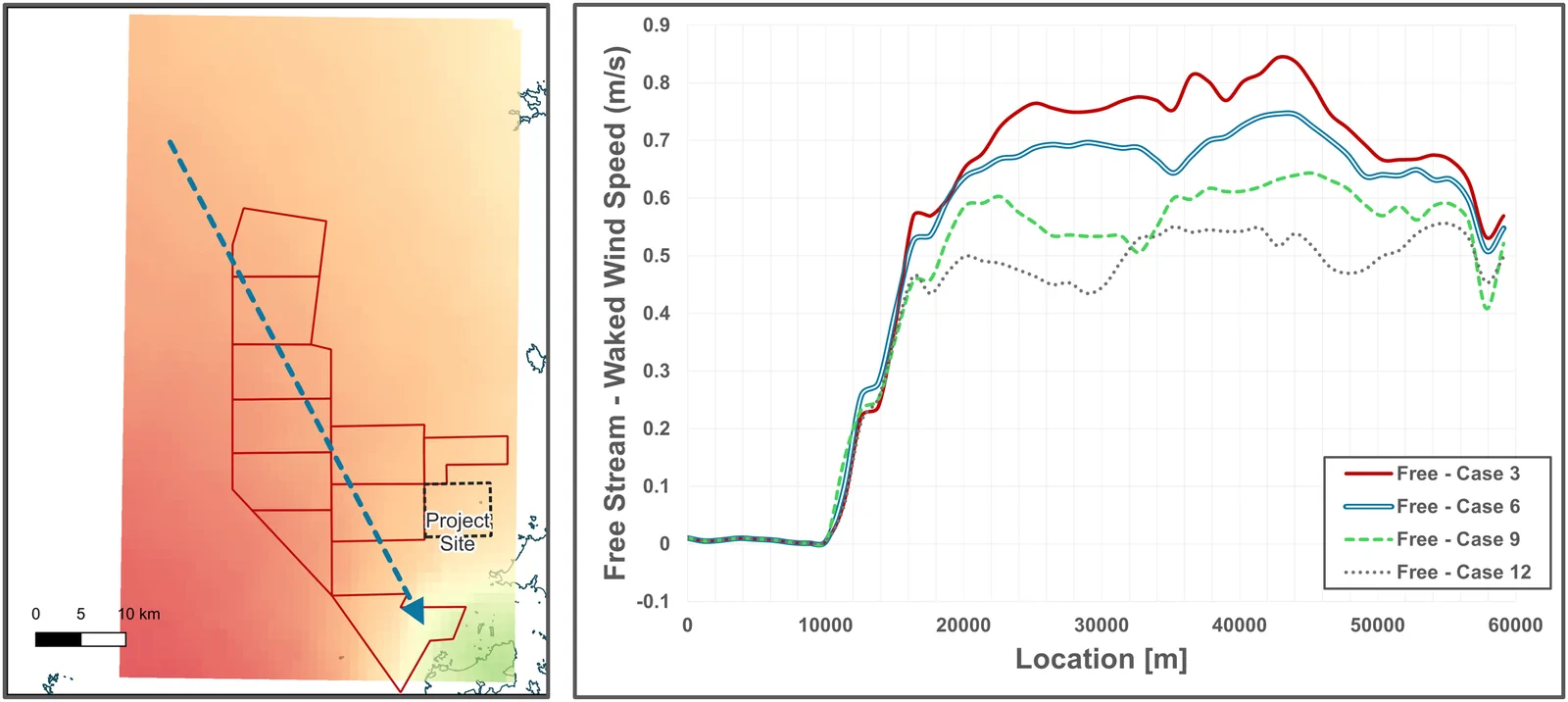
Cross-sectional spatial variation of the wind speed deficit (free-stream wind speed minus wake-effected wind speed) at hub height (150 m) along the blue arrow transect indicated in the Shinan cluster map. (a) The cross section is oriented along the prevailing wind direction approximately, traversing most wind farm zones. (b) Each curve represents the spatial profile of wind speed deficit for different inter-farm spacing scenarios, quantifying the impact of cluster wake interactions on available wind resource. Red solid line (Free-Sc 3), Blue double line (Free-Sc 6), Green dashed line (Free-Sc 9), Grey dotted line (Free-Sc 12).

The observed phenomenon where tighter spacing exacerbates wake-induced wind speed deficits can be primarily attributed to the physics of wake formation and recovery in clustered wind farm environments. Turbulent wakes generated behind wind turbines lead to a low-momentum region with reduced wind speeds and heightened turbulence, which slowly recovers with downstream distance due to turbulent mixing and ambient flow entrainment. When wind farms are positioned with minimal separation, these wakes overlap and interact, producing cumulative deficits that reduce available wind energy for downstream turbines, thereby impacting energy yield.

## Energy loss simulation results

Table 6 summarizes the sensitivity analysis results for a hypothetical offshore wind project site, evaluating the impact of inter-cluster distances and cluster site operational ratios on external wake losses. For each scenario, internal and external wake loss rates, total loss rate, P50 AEP, P50 capacity factor (CF), and the corresponding P90 AEP and P90 CF (estimated using a fixed P90/P50 ratio of 0.9, as described in Section 4.1) are presented.

**Table 6:** The summary of preliminary EYA results for each external wake case. P90 AEPs for each case were roughly estimated from P50 AEPs applying P90/P50 ratio of 0.9 without detailed independent uncertainty assessment due to absence of on-site measurement data.

Case	Dist. [km]	Operation [%]	Gross yield [GWh/y]	Internal wake loss [%]	External wake loss [%]	Total wake losses [%]	Total losses [%]	P50 yield [GWh/y]	P50 Capacity factor [%]	P90 yield* [GWh/y]	P90 Capacity factor* [%]
Base	–	–	1392.5	12.4	–	12.4	21.9	1,087.2	31.8	978.5	28.6
1	0	30	1392.5	12.4	3.7	15.6	24.8	1,047.5	30.7	942.7	27.6
2	0	60	1392.5	12.4	5.0	16.8	25.9	1,032.4	30.2	929.2	27.2
3	0	100	1392.5	12.4	8.0	19.4	28.1	1,000.5	29.3	900.5	26.4
4	1	30	1392.5	12.4	3.1	15.1	24.3	1,054.1	30.9	948.7	27.8
5	1	60	1392.5	12.4	4.4	16.3	25.3	1,039.6	30.4	935.6	27.4
6	1	100	1392.5	12.4	6.6	18.2	27.1	1,015.6	29.7	914.0	26.8
7	3	30	1392.5	12.4	2.2	14.3	23.6	1,063.8	31.1	957.4	28.0
8	3	60	1392.5	12.4	3.4	15.4	24.6	1,050.1	30.7	945.1	27.7
9	3	100	1392.5	12.4	5.2	17.0	26.0	1,031.0	30.2	927.9	27.2
10	5	30	1392.5	12.4	1.6	13.9	23.2	1,069.4	31.3	962.5	28.2
11	5	60	1392.5	12.4	2.8	14.9	24.1	1,056.6	30.9	950.9	27.8
12	5	100	1392.5	12.4	4.3	16.2	25.2	1,041.0	30.5	936.9	27.4

* P90 AEP and P90 CF were assumed from general assumption of P90/P50 ratio of 0.9.

The analysis revealed that external wake losses across scenarios ranged from 1.6% to a maximum of 8.0%, with total loss rates varying accordingly. The greatest external wake loss was observed in Case 3, where no inter-cluster distance is considered and all cluster sites are operational. For full operational scenarios (100% ratio), increasing the inter-cluster distance from 0 km (Case 3), to 1 km, 3 km, and 5 km led to reduced external wake losses of 8.0%, 6.6%, 5.2%, and 4.3%, respectively. This trend demonstrates that longer horizontal separations allow more room for wake recovery, substantially mitigating external wake impacts.

When considering the cluster operational ratio, with no specified inter-cluster distance, external wake losses increased sharply with higher ratios: 3.7% (30%), 5.0% (60%), and 8.0% (100%), compared to the Base case without external wakes. Correspondingly, P50 capacity factor differences reached up to 2.5% between scenarios.

Using the fixed P90/P50 ratio of 0.90 without independent uncertainty analysis, the estimated P90 capacity factor for each scenario was approximately 3.0% lower than the P50 value. In Case 3, compared to the Base case, the capacity factor suffered a 2.2% reduction and P90 annual energy yield decreased by roughly 8.0% due to external wakes.

Overall, the results indicate that greater completion and operational rates of neighbouring offshore projects, as well as narrower distances between clusters, can significantly increase the annual energy yield losses attributable to external wakes. Offshore wind clusters within close geographic proximity demand careful consideration of external wake effects during yield assessments. Additionally, composite factors including the construction status, operational period, and turbine types of adjacent clusters should be thoroughly incorporated during the design and operational planning phases. External wake losses should be recognized not only for their impact on annual energy production, but also for their broader negative influence on project financial and contractual stability.

## Economic loss simulation results

### SMP and REC scenarios

To estimate the economic impact of external wake losses for a hypothetical offshore wind project in the Shinan cluster, both the System Marginal Price (SMP) and Renewable Energy Certificate (REC) values were considered. RECs certify the environmental value of electricity produced from renewable sources and are issued to large-scale producers under Korea’s Renewable Portfolio Standard (RPS), enabling them to fulfil renewable energy supply obligations.

In fixed-price contract competitive bidding, long-term (20-year) contracts are awarded based on the combined price of SMP and REC (or REC price adjusted by weighting factors), negotiated between power producers and obligated suppliers. According to the revised RPS management guidelines (July 2021), coastal offshore wind is assigned a base REC weight of 2.0 (tidal flat or seawall), while offshore wind receives a base weight of 2.5, with final composite weightings determined by site-specific factors (such as transmission distance and water depth). Project revenue is calculated by multiplying actual energy output by the combined SMP and REC prices; any reduction in output (due to external wake losses, for example) directly reduces economic returns.

Therefore, evaluating economic loss sensitivity through combinations of SMP and varying REC weights is a critical tool for assessing project feasibility and financial security in offshore wind development. This study analysed changes in annual energy revenues, under different SMP + REC scenarios, to quantify long-term economic impacts attributable to external wake effects.

Reference values were drawn from the ‘2025 Korea Offshore Wind Fixed-Price Contract Competitive Bidding Announcement (first half)’, which set an upper price limit for land-based projects at 176,565 KRW/MWh (SMP + 1 REC). The reference SMP for land was 86,350 KRW/MWh, yielding a REC price of 90,215 KRW. Economic loss sensitivity was assessed for several REC weights (2, 2.5, 3.5, and 4.5). These scenarios cover basic weights (coastal: 2, offshore: 2.5) and the maximum expected REC weighting for deep water, remote floating wind clusters, such as Ulsan, which could reach 4.5 based on local depth and transmission distance. In Section 4.3.2, economic losses are compared for each combination of SMP and REC unit price and weighting factor, quantifying the annual energy yield impacts of external wake losses and providing concrete evidence for domestic offshore wind developers to support project feasibility decisions.

### Economic loss sensitivity analysis

Table 7 summarizes the results of economic loss sensitivity analysis based on annual energy shortfall simulations caused by external wakes. The analysis focuses on P90 annual energy yield, as this conservative metric is typically used during financial structuring and debt due diligence for wind projects. Assessing losses based on P90 more accurately reflects the true financial risk and potential economic impact to investors and lenders.

**Table 7:** Summary of sensitivity analysis on economic losses based on P90 AEP due to external wake losses, comparing four (4) SMP and REC scenarios (2.0, 2.5, 3.5, 4.5 RECs). All economic losses are presented as the difference from the Base case which was estimated without external wake effects.

Case	Dist. [km]	Operation [%]	External wake loss [%]	P90 yield [GWh/y]	P90 CF [%]	AEP change [%]	Economic losses for each SMP, REC scenario [0.1 billion KRW/year](* Million $ with currency of 1500 KRW/1 USD)			
							SMP + 2.0 REC	SMP + 2.5 REC	SMP + 3.5 REC	SMP + 4.5 REC
Base	–	–	–	978.5	28.6	Base	Base	Base	Base	Base
1	0	30	3.7	942.7	27.6	− 3.7	− 95.4(−6.4)	− 111.6(−7.4)	− 143.9(−9.6)	− 176.1(−11.7)
2	0	60	5.0	929.2	27.2	− 5.0	− 131.6(−8.8)	− 153.9(−10.3)	− 198.4(−13.2)	− 242.9(−16.2)
3	0	100	8.0	900.5	26.4	− 8.0	− 208.3(−13.9)	− 243.5(−16.2)	− 313.9(−20.9)	− 384.3(−25.6)
4	1	30	3.1	948.7	27.8	− 3.1	− 79.7(−5.3)	− 93.1(−6.2)	− 120.1(−8.0)	− 147.0(−9.8)
5	1	60	4.4	935.6	27.4	− 4.4	− 114.5(−7.6)	− 133.9(−8.9)	− 172.6(−11.5)	− 211.3(−14.1)
6	1	100	6.6	914.0	26.8	− 6.6	− 172.1(−11.5)	− 201.2(−13.4)	− 259.4(−17.3)	− 317.6(−21.2)
7	3	30	2.2	957.4	28.0	− 2.2	− 56.3(−3.8)	− 65.8(−4.4)	− 84.8(−5.7)	− 103.9(−6.9)
8	3	60	3.4	945.1	27.7	− 3.4	− 89.2(−5.9)	− 104.3(−7.0)	− 134.4(−9.0)	− 164.6(−11.0)
9	3	100	5.2	927.9	27.2	− 5.2	− 135.0(−9.0)	− 157.8(−10.5)	− 203.4(−13.6)	− 249.1(−16.6)
10	5	30	1.6	962.5	28.2	− 1.6	− 42.8(−2.9)	− 50.1(−3.3)	− 64.6(−4.3)	− 79.1(−5.3)
11	5	60	2.8	950.9	27.8	− 2.8	− 73.6(−4.9)	− 86.0(−5.7)	− 110.9(−7.4)	− 135.8(−9.1)
12	5	100	4.3	936.9	27.4	− 4.3	− 111.0(−7.4)	− 129.8(−8.7)	− 167.3(−11.2)	− 204.8(−13.7)

For the hypothetical offshore wind project considered (as spatially described in Fig 2), the average water depth is below 20 m and grid connection distance is approximately 20 km, so the REC weighting is not expected to exceed 3.5. However, a range of REC weighting scenarios were evaluated to enable project developers to quantitatively understand the range of potential losses. The results indicate that, under Case 3 (representing the most significant external wake impact) annual economic losses reach 20.83 billion KRW at a REC weight of 2, and escalate to 38.43 billion KRW at a REC weight of 4.5. Assuming a conservative 20-year project life, this could result in a maximum cumulative loss of 770 billion KRW.

These findings are consistent with Ryu et al. (2025), which cited that Orsted's Hornsea project submitted documentation to the UK government indicating potential compensation claims ranging from €5.5 million to €199 million (approx. 9–32.3 billion KRW at 1 EUR = 1,630 KRW) over its operational period due to external wakes from the Outer Dowsing project, even though the average separation was 20–30 km. In this study's Case 3 (with no inter-farm separation) the potential losses are reasonably expected to exceed those referenced in the UK case.

In Case 12 (5 km inter-cluster distance, 100% cluster operation), annual losses increase from 12.98 billion KRW (REC weight 2.5) to 16.73 billion KRW (REC weight 3.5) and 20.48 billion KRW (REC weight 4.5), but are reduced by roughly 47% compared to Case 3, demonstrating the benefits of adequate project spacing.

Overall, annual energy shortfalls due to external wakes have profound implications for project long-term profitability and financial stability. This is especially important as the trend toward large, closely clustered offshore wind zones can lead to direct conflicts of interest between multiple developers. Thus, policy and regulatory responses including compensation frameworks, minimum inter-project distance rules, and the adoption of technical mitigation measures are essential subjects for further discussion and implementation.

## Conclusion and implications

This study quantitatively analysed the impact of inter-cluster distance and external wake effects on energy yield and project economics, using the Shinan offshore wind cluster as a case study amid expanding government-led offshore wind cluster development. Sensitivity analysis across 12 scenarios showed that increasing the inter-cluster spacing from 0 km to 1 km, 3 km, and 5 km reduced external wake-induced energy losses from –8.0% to –6.6%, –5.2%, and –4.3%, respectively, with corresponding annual revenue loss reductions of up to 14.66 billion KRW (comparing Cases 3 and 12 at REC 3.5). These results highlight the need for policy measures such as minimum separation guidelines, which can mitigate profit loss and minimize potential disputes between project developers.

Currently, Korea’s offshore wind permitting and design guidelines lack institutional consideration for external wake effects. However, as large-scale cluster development becomes reality, coordinating interests and establishing compensation frameworks among developers is becoming increasingly important. Based on the findings, the study proposes three key legal and institutional improvements:

First, mandatory submission of external wake impact documentation. In the UK, cumulative impact assessments (CIA) are required for offshore wind projects [45]: these evaluate the overlapping impacts of individual projects on the environment and local communities, considering past, present, and planned activities. By systematically stating external wake impact assessment requirements following Europe’s precedent objective information would support fair review, permitting, and resolution among developers, and help prevent legal or commercial disputes [46,47]. For example, requiring government-mandated assessments when other permitted or operating projects exist within a specified radius (e.g., 20–30 km) would improve transparency, accountability, and reliability.

Second, expanding R&D and developing domestic standards and models for objective quantification of external wakes and their cumulative impact. Enhancing research projects, field data sharing, and the creation of advanced predictive models at the national level would empower stakeholders to assess losses accurately and reduce conflict risk during site selection, contributing to robust industry confidence.

Third, establishing minimum inter-cluster distances at the planning stage. Instituting clear minimum separations between offshore wind farms is critical for managing external wakes, ensuring economic viability, and building trust in the market. Insufficient separation exacerbates cumulative wakes, energy losses, and turbine fatigue, while well-defined rules support coexistence with local communities and various maritime interests. Reviewing international standards (see Section 2.2) and this study’s sensitivity results would help define a comprehensive Korean offshore wind separation policy from the outset, fostering sustainable national sector growth.

According to Tamarindo, a global renewable energy network, offshore wind projects facing legal or commercial disputes over external wake impacts in the UK are expected to exceed 20 GW of capacity by 2025. Economic compensation for losses from external wakes is a likely issue in Korea’s clustered offshore wind market. Preventive strategies require sufficient project spacing, active cluster control, and, at the policy design stage, follow-up research on uncertainty, risk assessment, and scenario-based financial impact analysis under various siting and meteorological conditions.

This paper provides an empirical foundation for a sustainable Korean cluster-based offshore wind planning model and supports the design of fair, transparent stakeholder frameworks, offering strategic insights for both academic research and future offshore wind policy and development.

## Limitations and future work

This study quantitatively assessed the impact of external wake effects on energy production and economic performance, focusing on high-resolution mesoscale modeling of meteorological conditions, operational ratios of individual wind farms, and inter spacing scenarios within the Jeollanam-do Shinan offshore wind cluster zone. Nevertheless, the research presents several limitations and potential directions for further development.

First, the numerical modeling results are inherently dependent on the specific meteorological and wind conditions of the Shinan offshore region such as annual mean wind speed, prevailing wind direction, and turbulence intensity. Therefore, the spatial patterns and magnitude of external wake losses may differ under distinct atmospheric environments or in other offshore areas. Future research should comprehensively evaluate the sensitivity of wake effects to variations in meteorological parameters, including atmospheric stability, boundary-layer structure, and vertical wind speed profiles. Such investigations are essential to reduce the uncertainty between simulated outcomes and actual conditions. Continuous comparison and validation between measured power production data and model results under diverse environmental conditions are required to ensure the representativeness and generalizability of the findings.

Second, while increasing inter-farm spacing can effectively mitigate external wake losses, it inevitably introduces drawbacks such as inefficient use of marine space, additional infrastructure requirements (e.g., transmission and grid connection), and higher capital expenditures associated with auxiliary civil works. Hence, a quantitative assessment is needed to identify the trade-off between the energy gains achieved from wake loss reduction and the economic costs arising from expanded spatial and infrastructure demands. In particular, attention should be given to potential diminishing returns, where the reduction of wake-induced energy loss becomes marginal beyond a certain separation distance. Deriving the optimal spacing range that maximizes overall techno-economic and societal benefits represents a crucial task for both policy formulation and industry practice.

Third, to enhance the sustainability and international competitiveness of large-scale offshore wind cluster developments in Korea, it is essential to establish a national framework for minimum inter-farm spacing and external wake impact assessment that reflects key influencing factors such as hub-height-dependent wake expansion characteristics, cluster radius, and cumulative operational years. This effort requires the acquisition of empirical wake validation datasets and the development of predictive and compensatory models through coordinated national R&D programs. Furthermore, policymakers should recognize the importance of applying differentiated criteria and location-specific optimization to ensure that such standards deliver strategic and equitable benefits across regional and industrial contexts.
